# Impact of Diet-Modulated Butyrate Production on Intestinal Barrier Function and Inflammation

**DOI:** 10.3390/nu10101499

**Published:** 2018-10-13

**Authors:** Knud Erik Bach Knudsen, Helle Nygaard Lærke, Mette Skou Hedemann, Tina Skau Nielsen, Anne Krog Ingerslev, Ditte Søvsø Gundelund Nielsen, Peter Kappel Theil, Stig Purup, Stine Hald, Anne Grethe Schioldan, Maria L. Marco, Søren Gregersen, Kjeld Hermansen

**Affiliations:** 1 Department of Animal Science, Aarhus University, 8830 Tjele, Denmark; hellen.laerke@anis.au.dk (H.N.L.); mette.hedemann@anis.au.dk (M.S.H.); tinas.nielsen@anis.au.dk (T.S.N.); annek.ingerslev@anis.au.dk (A.K.I.); ditteg.nielsen@anis.au.dk (D.S.G.N.); peter.theil@anis.au.dk (P.K.T.); stig.purup@anis.au.dk (S.P.); 2 Department of Hepatology and Gastroenterology, Aarhus University Hospital, 8000 Aarhus, Denmark; Stine.Hald@aarhus.rm.dk; 3 Department of Endocrinology and Internal Medicine, Aarhus University Hospital, 8000 Aarhus, Denmark; anneschioldan@gmail.com (A.G.S.); soeren.gregersen@aarhus.rm.dk (S.G.); kjeld.hermansen@clin.au.dk (K.H.); 4 Department of Food Science and Technology, University of California Davis, Davis, CA 95616, USA; mmarco@ucdavis.edu

**Keywords:** dietary fibre, butyrate, gut barrier function, gut inflammation, systemic inflammation

## Abstract

A major challenge in affluent societies is the increase in disorders related to gut and metabolic health. Chronic over nutrition by unhealthy foods high in energy, fat, and sugar, and low in dietary fibre is a key environmental factor responsible for this development, which may cause local and systemic inflammation. A low intake of dietary fibre is a limiting factor for maintaining a viable and diverse microbiota and production of short-chain fatty acids in the gut. A suppressed production of butyrate is crucial, as this short-chain fatty acid (SCFA) can play a key role not only in colonic health and function but also at the systemic level. At both sites, the mode of action is through mediation of signalling pathways involving nuclear NF-κB and inhibition of histone deacetylase. The intake and composition of dietary fibre modulate production of butyrate in the large intestine. While butyrate production is easily adjustable it is more variable how it influences gut barrier function and inflammatory markers in the gut and periphery. The effect of butyrate seems generally to be more consistent and positive on inflammatory markers related to the gut than on inflammatory markers in the peripheral tissue. This discrepancy may be explained by differences in butyrate concentrations in the gut compared with the much lower concentration at more remote sites.

## 1. Introduction

A major challenge in most affluent societies is the almost epidemic growth in obesity and metabolic syndrome (MetS) [1,2]. MetS is a cluster of interrelated metabolic abnormalities including insulin resistance, hyperglycemia, and hyperlipidemia. Obesity is the pre-stage that leads to MetS but the central feature of this condition is insulin resistance that greatly increases the risk of the development of type 2 diabetes, cardiovascular diseases, and liver dysfunction [1,2]. A high intake of energy dense, high-fat, high-sugar, and low dietary fiber foods is a key factor responsible for this development [3,4]. A consequence of chronic over nutrition is accumulation of fat in adipose tissue, which subsequently becomes infiltrated by immune cells [5,6]. This condition causes low-grade inflammation that gives rise to a mild and sustained increase in immune mediators such as acute-phase C-reactive protein (CRP), interleukin (IL)-6, and tumor necrotic factor (TNF)-α in the systemic circulation [5,7,8]. The same dietary factors responsible for the development of obesity and MetS are also important risk factors for gut inflammation and the development of colorectal cancers and inflammatory bowel diseases [9,10,11].

Fermentable dietary fibre (DF) represent the fraction of the food not digested in the small intestine with the advent of endogenous enzymes [12] but can be converted into an array of small organic metabolites by the microbiota in the large intestine, the most important being the short-chain fatty acids (SCFA), acetate, propionate, and butyrate [13]. The low intake of DF in affluent societies is a limiting factor for the maintenance of a viable and diverse microbiota and the production of SCFA [14,15,16]. In this context, suppressed production of butyrate plays a major role in colonic health and function [9,10]. Butyrate is a C-4 fatty acid and the third most abundant SCFA in the gut of all mammals [17,18]. The concentration in the gut and the circulation can, however, be modulated primarily via the content and composition of DF [12,19]. Apart from being the preferred fuel for the colonic epithelial cells [20] and the major regulator of cell proliferation and differentiation [21,22,23], butyrate has also been shown to exert important actions related to cellular homeostasis such as anti-inflammatory, antioxidant and anti-carcinogenic functions [24,25,26]. Central to the biological effects of butyrate in the gut as well as in the periphery is the influence of butyrate as a histone deacetylase inhibitor and its binding to several G protein-coupled receptors [23,27,28]. 

While there have been several recent papers addressing the impact of an altered microbiota on gut and metabolic health [29,30,31], the present paper will mainly focus on the interaction between butyrate and markers for gut and metabolic health. Core data for the paper derive from the Danish Strategic Research-funded project: “Concepts for enhanced butyrate production to improve colonic health and insulin sensitivity”—an interdisciplinary project using both in vitro and in vivo models (pigs and humans) to study the gut and metabolic implications of enhanced butyrate production.

## 2. The Intestinal Tract and Its Microbiota 

Specialized epithelial cells constitute the barrier surfaces that separate the mammalian host from the external environment [32]. The gastrointestinal tract epithelium is the largest of these barriers that segregate the microbiota from the interior by physical and biochemical means [32]. The intestinal epithelium is one layer thick and its cellular fitness is maintained through frequent cycles of apoptosis and renewal [33]. On top of the epithelium is the mucus layer that also contributes to the protection of the epithelium from the microbiota in the gut lumen. The principal component of the colonic mucus is the homo-oligomerized mucin gel-forming glycoprotein (MUC2) secreted by goblet cells [34]. The goblet cells continuously renew the mucus layer. The mucus also contains anti-microbial peptides and IgA, which both serve to limit the number of bacteria that reach the host epithelium [29]. 

The human intestinal tract contains a diverse collection of micro-organisms, encompassing bacteria, archaea, fungi and virusses [14,35]. The numbers of microorganisms can reach 10^11^–10^12^ per gram; tenfold greater than the number of cells in the human body [14,35]. More than 50 genera and 400–1000 species of bacteria have been found in human feaces [36,37]. Butyrate-producing species in the intestine are predominantly found in the Firmicutes phylum, and most specifically the Ruminococcaceae, and Lachnospiraceacae families [16,38]. These families encompass *Faecalibacterium prausnitzii* in the *Clostridium leptum* cluster (Clostridial cluster IV) and *Eubacterium rectale/Roseburia* spp. in the *Clostridium coccodies* (Clostridial cluster XIVa) cluster of *Firmicutes* [16,38]. In addition to these groups, butyrate-producing bacteria are widely distributed across several clusters [16,38].

Certain bacteria, i.e., the mucosa-associated bacteria possess the ability to bind to, interact with, and metabolize mucins. In contrast to luminal bacteria, mucosa-associated bacteria are more likely to be in close contact with the intestinal barrier than luminal bacteria as they have access to additional nutrition sources of the intestinal mucus layer [39]. Mucin degradation is part of the normal intestinal cell turnover but excessive degradation may occur when a DF-deficient diet, e.g., a Western-style diet, is consumed [40]. Under those conditions the microbiota may switch from degradation of external substrates to endogenous substrates [33,39]. 

## 3. Dietary Factors Modulating the Microbiota and Butyrate Production

**Figure 1:**
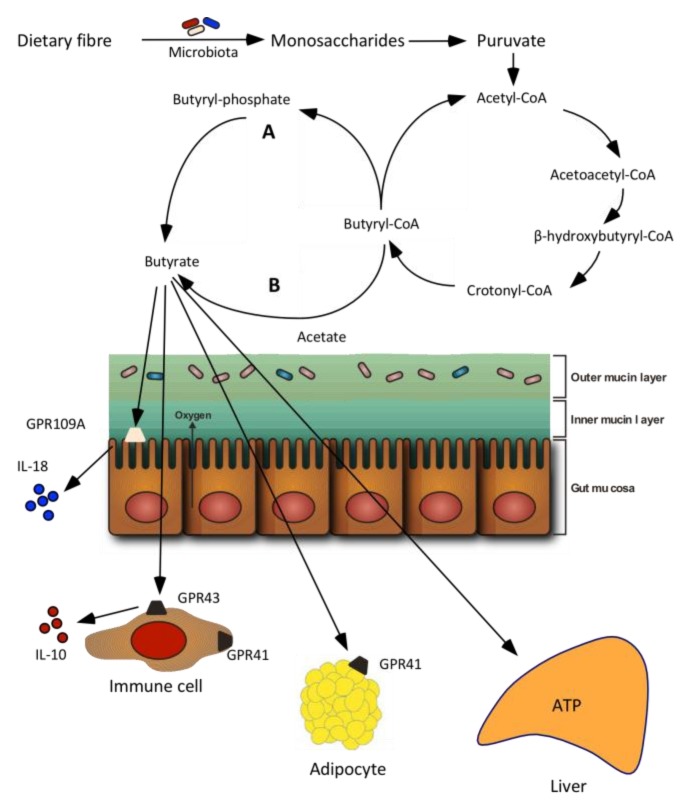
Butyrate formation from dietary fibre and absorption in the large intestine. Two pathways of butyrate production from butyryl-CoA in bacteria has been reported. Letter “A” indicates that butyryl-CoA is phosphorylated to butyryl-phosphate and converted to butyrate via butyrate kinase. Letter “B” shows that the CoA moiety of butyryl-CoA is transferred to external acetate via butyryl-CoA: acetate transferase, leading to the formation of butyrate and acetyl-CoA [38]. Several receptors for butyrate including G-protein-coupled receptors 41 (GPR41), GPR43 and GPR109A have been identified. GPR41 is found in adipose tissues and immune cells, GPR43 in immune cells whereas GPR109A is present in colonic cells. GPR109A is essential for butyrate-mediated induction of IL-18 in colonic epithelium. Modified from [27].

The large intestine is the main site for SCFA production [12,40]. The environment in the large intestine has all the conditions for prolific bacterial growth: warm, moist, anaerobic, and filled with feed residues that flow at a relatively low speed. A common feature of the colonic bacteria is their diverse repertoire of catabolizing enzymes and metabolic capabilities that with all measures is much higher than that of their host [41]. Combined with the long retention time and moist environment in the large intestine these conditions are favourable for the degradation of even very complex DFs [41]. Butyrate is produced from carbohydrates via glycolysis from the combination of two molecules of acetyl-CoA to form acetoacetyl-CoA, followed by stepwise reduction to butyryl-CoA (Figure 1) [42]. There are two different pathways for the final step in butyrate formation from butyryl-CoA. In the first pathway, butyryl-CoA is phosphorylated to form butyryl-phosphate and subsequently transformed to butyrate via butyrate kinase. In the second pathway, the CoA moiety of butyryl-CoA is transformed to acetate via butyryl-CoA: acetate CoA-transferase leading to the formation of butyrate and acetyl-CoA [42]. Glutamate, lysine, histidine, cysteine, serine, and alanine can also lead to butyrate formation [38].

The most important determinants of SCFA production are the amounts and types of residues that enter the large intestine [41,43]. The main substrates available for fermentation are non-starch polysaccharides, various forms of resistant starch, and non-digestible oligosaccharides, sugar alcohols, and proteins [41]. Also host-produced substances such as glycoproteins, exfoliated epithelial cells, and pancreatic secretions contribute [41]. The non-digested carbohydrates are exposed to the action of the hydrolytic bacteria, which produce extracellular cellulases and other enzymes that degrade the polysaccharides to oligosaccharides [40]. Spatial analyses have shown an uneven distribution of the bacterial communities to the solid compared to the liquid phase. Analysis of PCR-amplified 16S rRNA sequences have revealed a significant lower percentage of Bacteroidetes and a slightly higher percentage of *Firmicutes* among bacterial associated to particles compared to liquid [44]. In a study of Walker et al. [45] the significant association with solid particles was found for relatives of *Ruminococcus*-related clostridial cluster IV species that include *Ruminococcus flavefaciens* and *R. bromii*, which together accounted for 12.2% of particle-associated, but only 3.3% of liquid phase, sequences. The produced oligosaccharides are either used directly by the hydrolytic bacteria or cross-fed to non-hydrolytic bacteria that convert the carbohydrate monomers (pentoses and hexoses), through a variety of intermediates to SCFA, mostly acetate, propionate, and butyrate [16,44,46]. The diet affects and interacts with the microbial community and, thereby, influence the metabolic outcome through several interrelated mechanisms. Firstly, the metabolism is regulated within each individual species of gut bacterium where alternative substrates can give rise to different products because of fermentation via different metabolic routes. Secondly, the same substrate can be processed via different routes depending on the supply rate or the physiology and environment of the bacterial cell [46,47].

The chemical composition and physicochemical properties of the dietary carbohydrates influence the amount and composition of SCFAs produced during fermentation. This has been found in vitro when fermenting pure polysaccharides, various fibres, and ileal effluents from humans and pigs, and in animal studies where diets varying in DF content and composition have been used (for overview see reference [12]). Substrates stimulating the formation of butyrate are starch, arabinoxylan-rich whole grains, and brans from cereals such as wheat, rye and oats. In contrast, cellulose, xylan, pectin and pectin-rich fractions in general all result in relatively low formation of butyrate [12,43,48]. In recent studies with intact pigs and portal vein catheterised pigs it was found that diets high in DF resulted in substantially higher SCFA large intestinal pool size and SCFA absorption than a low DF Western-style diet high in refined carbohydrates from sugar and refined wheat flour [19,49] (Table 1). The two high-DF diets applied in the studies were high in DF due to either resistant starch type 2 from raw potato and high-amylose maize starch (ratio 1:2) or ingredients high in arabinoxylan (whole grain rye and enzyme treated wheat bran) [19,49]. The relative higher increase in butyrate production following the arabinoxylan-rich diet was most likely caused by an effect of the substrate (arabinoxylan) and a relative increase in the abundance butyrate producing microorganims (i.e., *Faecalibacterium pausnitzii*, *Roseburia intestinalis*). The combined effect was a higher production of butyrate in the large intestine and net portal absorption with the arabinoxylan rich diet compared with the Western style diet and the diet high in resistant starch [19,49], the latter also being higher in terms of butyrate production than the Western-style diet. The higher production of butyrate that occurred with the consumption of the two high-fibre diets (RS and arabinoxylan) resulted in higher butyrate concentrations in the large intestine and in central and peripheral blood supply also (Table 1). 

In a follow-up intervention study with human subjects with MetS, we used the knowledge generated in the pig studies to design a healthy carbohydrate diet, which was compared with a Western-style diet regarding gut [50] and metabolic health parameters [51]. The Western-style diet was low in DF (17.6 g/kg dry matter) primarily deriving from cereal products based on refined flours. In the healthy carbohydrate diet, the DF content was increased by using whole grain cereals, enzyme-treated wheat bran, high-amylose maize starch, and raw potato starch (64.0 g/kg dry matter) [52]. The two diets provided most of the total intake of DF, 21 g/day for the Western-style diet and 68 g/day for the healthy carbohydratw diet. The intervention period lasted 4 weeks and faecal and gut samples were taken at run-in and at the end of the intervention period. The healthy carbohydrate diet resulted in higher faecal SCFA concentrations with acetate and butyrate particularly being increased. However, while butyrate-producing microorganisms (i.e., *Faecalibacterium pausnitzii*, *Roseburia intestinalis*) were stimulated by the arabinoxylan-rich diet in the pig study, *Bifidobacterium* was the only group enriched by the healthy carbohydrate diet in the human study. Since *Bifidobacterium* are predominant acetate producers [38], the higher butyrate concentrations in faeces after consuming the healthy carbohydrate diet was presumably caused by the increased acetate production which was converted into butyrate [53]. Furthermore, this study also indicated that the faecal butyrate level during the run-in period had a profound influence on the efficacy of the diet. Six out of 10 subjects that had an initial butyrate concentrations below the median responded by increased butyrate concentration in faeces, whereas only 2 out of 9 subject responded by increased butyrate concentration when the initial concentration was above the median [52]. 

## 4. Butyrate Absorption and Signaling

**Figure 2:**
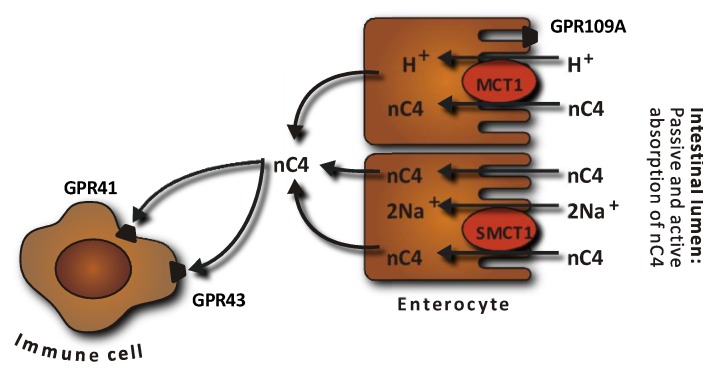
Absorption of butyrate (nC4) in the large intestine. Butyrate transport with monocarboxylate transporters (MCT) is saturable and coupled with H^+^ transport. Several G-protein-coupled (GPR) receptors for butyrate have been identified and detected in various tissues including the colonic epithelium (GPR109A) and immune cells (GPR41 and GPR43). SMCT, Na-coupled monocarboxylate transport. Modified from [54].

Two different mechanisms of SCFA absorption across the apical membrane of colonocytes are reported; diffusion of the undissociated form and active transport of the dissociated form by SCFA transporters (Figure 2) [22,54]. Butyrate has been found to increase the expression and activity of the active H^+^-coupled cotransporter (monocarboxylate cotransporter 1, MCT1) in cultured human colonic epithelial cells in concentration- and time-dependent manners [55]. Pig studies, demonstrated an increase in the MCT1 mRNA abundance and MCT1 gene expression in the caecum when butyrate production was increased by feeding diets high in resistant starch or arabinoxylan compared with control diets [56,57,58]. 

The SCFA-sensing G-protein-coupled receptors 41 (GPR41), GPR43 and GPR109A found on the intestinal epithelial cells are activated by SCFAs [28]. GPR41 and GPR43 are believed to provide a link between intestinal SCFA production and appetite and energy homeostasis [59] whereas GPR109A activates inflammation in colonic macrophages and dendritic cells, resulting in differentiation of regulatory T cells and IL-10-producing T cells [60,61]. The receptors have specificities for different SCFAs and butyrate binds to GPR41 to a greater extent than to GPR43 [62]. Stimulation of the GPR41 and GPR43 receptors is thought to promote the enteroendocrine secretion of peptide YY (PYY), which inhibits gastric emptying and intestinal transit time, thereby supressing appetite and by promoting glucagon-like peptide 1 (GLP-1), the latter with stimulatory effects on insulin secretion [63]. In the study reported in Table 1, higher arterial and portal vein concentrations of PYY and a delayed absorption of glucose were found after consumption of the DF-rich diet enriched in resistant starch. However, the higher PYY concentrations could not be related to a higher net portal appearance of either SCFA or butyrate [19,64]. Pig studies, wherein the expression of GPR41 and GPR43 have been quantified in the caecum and colon, also failed to demonstrate a direct link to intestinal butyrate production [57,58]. Instead, a rat study wherein fructooligosaccharides were used to stimulate butyrate production led to the finding of an increased density of GPR43 immunoreactive enteroendocrine cells in the proximal colon compared with a control diet [65]. GPR109A, another major GPR activated by butyrate, activates the inflammation-associated pathway in colonic macrophages and dendritic cells, resulting in differentiation of regulatory T cells and IL-10-producing T cells [60,61]. The secretion of IL-18 is also increased in intestinal epithelial cells via butyrate-stimulated signalling of GPR109A [66].

## 5. Butyrate and Intestinal Barrier Function

The barrier function of the epithelial cells is the first line of defence in the intestine [32]. Barrier integrity is largely the result of the proper functioning of tight junctions between the epithelial cells. Tight junctions consist of transmembrane proteins such as occludin, which seal the intercellular epithelial space, and plaque proteins e.g. zonula occludens-1 located on the intracellular side of the plasma membrane acting as an anchor to the transmembrane proteins [67]. The tight junctions control the diffusion of water, ions, and nutrients, while they restrict pathogen entry and thereby regulate permeability of the intestinal mucosal barrier [32]. The latter is of particular importance as studies have suggested that gut-derived endotoxin (lipopolysaccharide) might be crucially involved in the chronic inflammation observed in type 2 diabetes. Mice studies have shown that a high fat diet can increase the lipopolysaccharide content of the gut’s microbiota and result in metabolic endotoxaemia [68]; similar to what was found when lipopolysaccharides was infused subcutaneous for 4 weeks. A study with C57Bl/6 mice showed that a diabetic phenotype was associated with an increased gut permeability, endotoxaemia, and a specific gut microbial profile [69]. These preclinical findings in animals have been corroborated by clinical studies demonstrating that patients with MetS and type 2 diabetes exhibited endotoxaemia [70,71].

In vitro, sodium-butyrate at a concentration ranging between 1 to 10 mM has been found to significantly improve the epithelial barrier function in E12 human colon cells measured as *trans*-epithelial electrical resistance and permeability of fluorescein-isothiocyanate-dextran, whereas higher concentrations (50–100 mM) showed no beneficial effects [72]. These results are in accordance with a study using Caco-2 human intestinal cells that also showed improved barrier function at low butyrate concentration but reduced barrier function at excessive levels of butyrate [73]. The increased epithelial barrier function in E12 cells, however, was not manifested as increased expression of the intercellular junction protein, zonula occludens 1 but to the mucus produced by the goblet cells as indicated by lower expression of genes encoding for the gel-forming mucin (MUC2) at higher butyrate levels [72]. For Caco-2 cells, an earlier study has also failed to demonstrate regulation of occludin, claudin-1 and 4, or zonula occludens 1 gene expression by butyrate but in contrast to the mucin production in E12 cells, the reduced intestinal barrier function for Caco-2 cells appears to be related to increased intestinal epithelial cell apoptosis induced by butyrate at high concentration [74]. In pigs, diet-induced alterations in large intestinal SCFA production only showed minor influence on parameters related to intestinal barrier function. It was only the mRNA abundance of MUC2 that was influenced by the diet induced butyrate production [58] whereas the other parameters measured (murin 2, zonula occludens 1, and occludin) were not influenced. The relationship between *MUC2* expression and butyrate, however, was not directly related to either luminal concentration or pool size [58]. Other in vivo studies with rodents have also been ambiguous concerning the relationship between luminal butyrate (SCFA) levels and the abundance of MUC2 [75,76,77,78]. The transcription of the *MUC2* gene was negatively correlated with the butyrate pool in the caecum but no correlations between the *MUC2* transcription and SCFA were found in the colon [76]. In contrast, studies with isolated perfused rat colon [77,78] showed that MUC2 production and, thereby, indirectly *MUC2* gene expression, was increased at butyrate concentrations corresponding to the concentrations typically measured in the cecum and proximal colon of mammals [49]. In human subjects with MetS an increased colonic expression of MUC2 and tight junction protein occludin was observed following diet-induced increase in SCFA and butyrate production [50]. These latter results are in accordance with results from a pig study encompassing arabinoxylan [79] and from rodent and pig studies concerning resistant starch [80,81].

## 6. Butyrate and Inflammation

**Figure 3:**
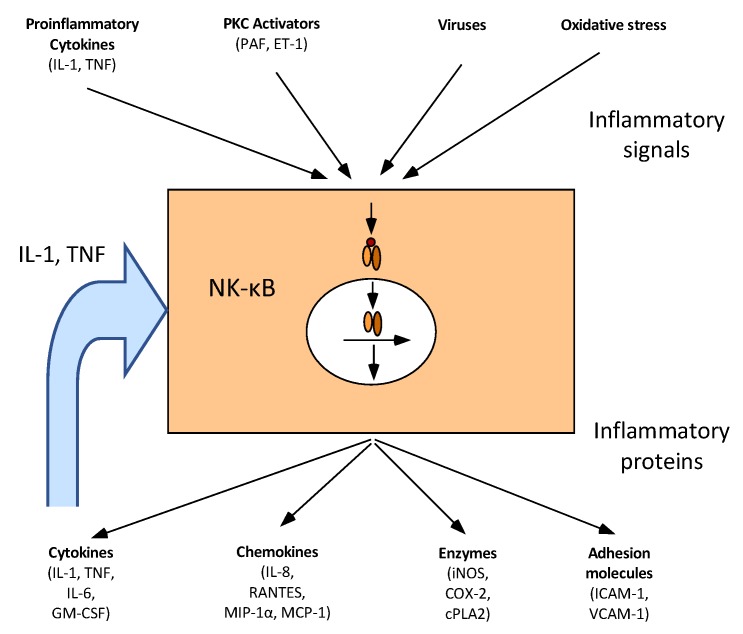
NF-κB central role in inflammation. Several pro-inflammatory signals such as cytokines, protein kinase C (PKC) activators, infectious agents or oxidative stress activates NF-κB. In response to these signals, NF-κB controls the expression of many mediators of the inflammatory reaction: cytokines, chemokines, enzymes and adhesion molecules. Modified from [84].

Inflammation is a normal defence mechanism that protects the host from infection and other insults. When an inflammatory response occurs, it is normally well-regulated to prevent excessive damage to the host, as well as self-limited and resolves rapidly. The self-regulation involves the activation of negative feedback mechanisms such as the secretion of anti-inflammatory cytokines, inhibition of pro-inflammatory signal cascades, the shedding of receptors for inflammatory mediators, and activation of regulatory cells [82]. A key point for these processes is the activation of transcription factor NF-κB. NF-κB is a key transcription factor that controls the expression of genes encoding proinflammatory cytokines, chemokines, inducible inflammatory enzymes such as inducible nitric oxide synthase and cyclo-oxygenase-2, adhesion molecules, growth factors, some acute phase proteins, and immune receptors (Figure 3) [83,84,85]. However, when inflammatory responses become excessive, it causes irreparable damage to host tissues leading to disease [5,86]. Inflammation can occur locally in the gut, where its chronic form may lead to inflammatory bowel disease, autoimmune disease, and cancer [33,39,87], or systemically, where chronic low-grade inflammation is associated with increased risk of insulin resistance, diabetes type 2, and atherosclerosis [5,7]. 

### 6.1. Intestinal Inflammation

Physical barriers, the epithelial cells, and biochemical barriers, the mucus, segregate the microbiota from mammalian immune cells in the intestine. These layers are essential to limit inappropriate inflammatory activations caused by diet, microbial metabolites, and bacteria [88,89]. An anti-inflammatory response to butyrate via NF-κB inhibition is reported from several in vitro and in vivo studies where decreased concentrations of myeloperoxidase, cyclo-oxygenase-2, adhesion molecules, and cytokines were identified [85,90,91,92]. An overview of human and animal in vivo studies is shown in Table 2. In our 4-week human intervention study with MetS subjects [52], the healthy carbohydrate diet caused a significantly lower mRNA abundance of monocyte chemotactic protein 1 (MCP1) and borderline reduction in IL-23A and NF-κB. Other genes (*cluster of differentiation 25* (*CD25*), *forkhead box P3* (*FOXP3*), *IL-10*, *interferon γ* (*INFγ*), *signal transducer and activator of transcription 3* (*STAT3*), *transforming growth factor β* (*TGFβ*), *tissue necrosis factor α* (*TNFα*)) were not influenced by the higher butyrate production [50]. The faecal calprotectin level was significantly reduced following the dietary treatment [50]. A reduced faecal calprotectin level has also been reported in a study where trans-galactooligosaccharides were used to raise the DF content [93]. This study also reported lower plasma CRP levels [93]. In contrast, in the study with intact pigs, where the large intestinal pool size of butyrate was 2.4 to 4.2-fold higher for the high fibre diets enriched in resistant starch rich and arabinoxylan, no significant effects on the expression of genes coding for MCP1 and TNFα or the proinflammatory nuclear transcription factor NF-κB were found [58]. In rats, colonic TNFα expression was found to be down-regulated following a diet containing 5% *P. ovata* seeds compared with control animals fed a low-DF diet [94], and a diet with 8% oligofructose-enriched inulin fed for 28 weeks, down-regulated the expression of TNFα and NF-κB in the colon [95]. In both of these studies, the fibre-enriched diets were associated with a higher colonic in situ production [94] and faecal concentration [95] of total SCFA and butyrate.

Butyrate and other SCFAs are also reported to bind and activate the nuclear transcription factor PPARγ [96] which antagonizes NF-*κ*β signal transduction causing an anti-inflammatory effect in the gut. In vitro, butyrate reduced inflammation by inhibition of NF-κB activation and up-regulated PPARγ expression in human HT-29 colonic epithelial cells [97]. Studies with mice [98], pigs [99] and humans [100] have indicated that the expression of the PPARγ is implicated in the pathology of numerous diseases including inflammatory bowel disease. Under normal physiological conditions the effects in different studies are variable with studies in pigs, showing both no change in intestinal PPARγ gene expression [58] and an upregulation in the expression of PPARγ [101]. Furthermore, colonic PPARγ expression was upregulated in mice chemically induced with inflammatory bowel disease following dietary intervention with 5% DF from resistant starch and soluble maize fibre [102].

Oxidative stress is part of the inflammation processes [9]. During oxidative stress, there is an imbalance between the generation of reactive oxygen species and the antioxidant defence mechanisms, leading to a cascade of reactions in which lipids, proteins and/or DNA may be damaged [25]. In vitro studies indicate that the antioxidant enzymes, superoxide dismutase-2 (SOD2) *a*nd catalase (CAT) were increased following treatment with a range between 50 to 100 mM butyrate, whereas no effects were found at levels of 0.1 to 10 mM [72]. This higher antioxidant capacity suggests an improvement in the level of oxidative stress in the cells. In a double blind, cross-over study with 16 healthy human volunteers, daily rectal administration of a 100 mM sodium butyrate enema for 2 weeks resulted in significantly higher glutathione and lower uric acid concentrations compared to placebo (saline) [25]. Changes in glutathione and uric acid were accompanied by increased and decreased expression, respectively, of their rate-limiting enzymes, whereas none of the other parameters were changed relative to the placebo [25]. A concentration of 100 mM of butyrate in the large intestine is far above the normal physiological levels in mammals [18,49] and can only be achieved by enema. Nielsen et al. [72] suggested that the absence of effects on the transcriptional levels at low concentrations of butyrate might be a consequence of regulation at the protein level [103] and that the low levels of the antioxidant enzymes expressed following treatment with butyrate might simply indicate the presence of a favorable balance between antioxidant enzymes and reactive oxygen species. 

### 6.2. Systemic Inflammation

**Figure 4:**
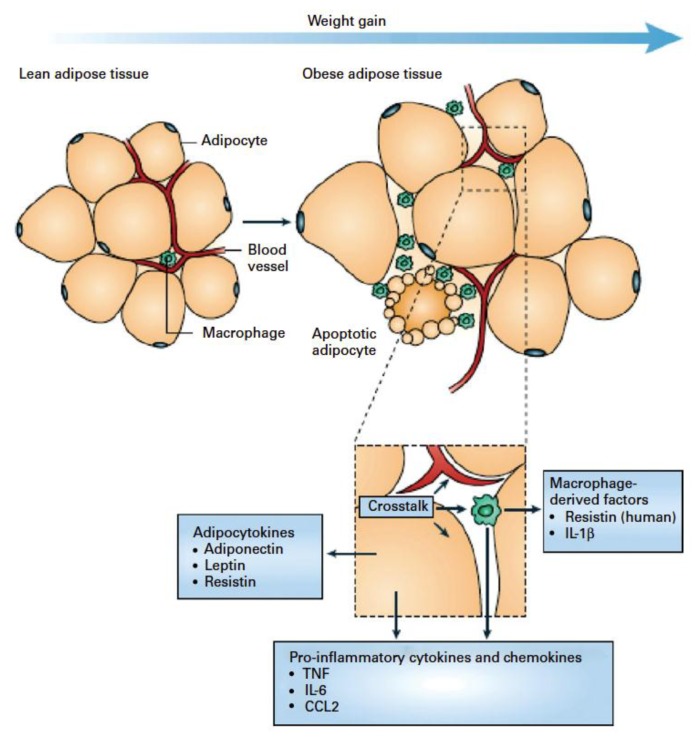
Schematic representation of the interaction between adipocytes and macrophages showing some of the molecules released. Expansion of adipose tissue during weight gain leads to the recruitment of macrophages through various signals (e.g. chemokines such as chemokine (C-C motif) ligand 2 (CCL2) released by adipocytes. Macrophages accumulate around apoptotic adipocytes. Mediators synthesized by adipocytes and resident macrophages contribute to local and systemic inflammation. Reproduced from [6].

The existence of inflammatory diseases in the gut has long been long recognized. However, only recently chronic low-grade systemic inflammation has received attention, particularly in relation to obesity, MetS, and cardiovascular disease [5,86]. It is known that chronic over-supply of energy leads to accumulation of fat in adipose tissue, which can then become infiltrated by immune cells (Figure 4) [5]. Observational studies have shown elevated blood levels of inflammation-associated markers in humans with incident type 2 diabetes or MetS [104,105]. The up-regulation of systemic indicators of inflammation include leucocyte count, blood concentrations of acute-phase proteins, pro-inflammatory cytokines, chemokines, soluble adhesion molecules, and prothrombotic mediators [5]. The up-regulation is usually modest, less than 2-fold above what is observed in controls and the concentrations converse in obesity and after weight loss [5]. Meijer et al. [88] have reviewed the molecular literature linking SCFA to systemic inflammation. They concluded that in spite of conflicting results, butyrate seems to have an anti-inflammatory effect mediated by signaling pathways like NF-κB and inhibition of histone deacatylase [88]. They concluded that the discrepancies in results found between different studies could be explained by different cell types and differences in their proliferative and differentitative status. The authors further pointed to the need to perform studies at relevant physiological concentrations, as some of the identified effects could be due to toxicity [88]. In the context of physiological concentrations, it is important to consider that butyrate is present in millimolar concentrations in the gut but only at micromolar concentrations in the periphery (Table 1) [19,49]. 

An inverse relationship between the intake of DF and plasma concentrations of CRP [106,107,108] and the proinflammatory cytokine IL-6 [109] have been found in prospective human studies. The results of short-term intervention studies, however, are ambiguous as shown in Table 3. It is also only few studies where the effects of the intervention diet on SCFA and butyrate concentrations in faeces or bloodstreams have been measured. However, it can be assumed that a higher intake of DF will increase SCFA and butyrate production as indicated by the data in Table 1 and the literature [12]. A human intervention study with healthy humans fed *trans*-galactooligosaccharides showed a significant increase in serum levels of the anti-inflammatory cytokine IL-10 and significant reductions in the levels of IL-6, IL-1β, and TNF-α [110]. In a study where, elderly subjects were given fructooligosaccharides, a decreased IL-6 mRNA expression in peripheral blood monocytes was observed [111] and a 12-week intervention with resistant starch to prediabetes subjects showed decreased TNF-α concentrations in plasma [112]. Intervention for 24 weeks with a healthy Nordic diet to subjects with features of MetS caused no change in the level of IL-1 Ra in plasma in contrast to the control diet where IL-1 Ra increased [113]. Analyses of the gene expression in abdominal subcutaneous adipose tissue showed that the healthy Nordic diet reduced inflammatory gene expression in the adipose tissue compared to the control diet [114]. Furthermore, a 5-week intervention study wherein arabinoxylan-rich rye was combined with oat and sugar beet fibre and fed to hypercholesterolemic subjects induced downregulation of high-sensitive-CRP (hs-CPR) [115]. Other intervention studies have been carried out with increased amount of fermentable substrate either by a combination of arabinoxylan and resistant starch [51] or the substitution of refined white flour, low in DF, with DF-rich whole grains [116,117] or supplemented with resistant starch [115,118]. These studies failed to demonstrate beneficial effects on either hs-CRP [116,117,119], IL-6 [116,117,118,119], IL-1Ra [117] or adiponectin [118,119] (Table 3). 

The studies mentioned have all dealt with chronic changes in concentration of inflammatory mediators. However, it should be noted that a rise in inflammation also takes place acutely following a meal [5]. Although the postprandial inflammatory responses only last for a few hours (4 h), it is repeated several times daily and may, therefore, probably also play an important role in the generation of insulin resistance. In healthy young individuals, an evening meal rich in indigestible carbohydrates from barley caused a higher plasma concentration of butyrate the following morning and prevented the glucose-induced postprandial rise in plasma IL-6 [120,121,122] and TFN-α [122]. In contrast, another study following the same dietary regime but with whole grain rye rather than barley failed to demonstrate any impact on inflammatory markers [123].

Taken together, diet-induced increases in butyrate production may influence intestinal and metabolic biomarkers of inflammation. However, the effects seem more consistent in the intestines than at the systemic level most likely reflecting the fact that the luminal concentration is approximately 1000-fold higher than in the peripheral system. It is also worth noticing that effects at the systemic level may require much longer intervention periods since an increased butyrate concentration acts on processes in cells and tissues typically developed over an extended period of time.

## 7. Conclusions

The production of butyrate in the gut and the concentrations in the gut and circulation can be modulated by dietary means, particularly through the content and composition of DF. There is good evidence that enhanced butyrate production may influence the gut barrier function and level of intestinal inflammation, whereas the effects on peripheral inflammation, in general, are less pronounced and more ambiguous. It can also be noted that inflammation markers in most studies are a secondary outcome and only one of the listed studies have checked for changes in SCFAs and butyrate in faeces. Therefore, more studies are warranted designed with peripheral inflammation as the primary end-point. Such studies could use specific ingredients known to stimulate butyrate and SCFA production but should also check the effect of diet on butyrate and SCFA in the gut and periphery.

## Figures and Tables

**Table 1:** Influence of diets varying in dietary fibre content and composition on short-chain fatty acids pool size in the large intestine and absorption of short chain fatty acids in intact and multi-catheterised pigs, respectively.

Diets	WSD	RSD	AXD
	Dietary composition, g/kg dry matter		
Total dietary fibre	72	186	196
Non-starch polysaccharides	58	55	144
Cellulose	29	34	37
Arabinoxylan	18	15	72
Resistant starch	6	113	8
Non-digestible oligosaccharides	2	5	29
	Pool size, mmol		
Total short-chain fatty acids	237 ^c^	512 ^b^	641 ^a^
Acetate	152 ^b^	320 ^a^	384 ^a^
Propionate	51 ^c^	109 ^b^	148 ^a^
Butyrate	19 ^c^	46 ^b^	79 ^a^
Branched-chain fatty acids	2.8 ^b^	3.8 ^a,b^	4.4 ^b^
	Absorption, mmol/day		
Total short-chain fatty acids	888 ^c^	1584 ^b^	2448 ^a^
Acetate	576 ^c^	960 ^b^	1488 ^a^
Propionate	197 ^c^	408 ^b^	576 ^a^
Butyrate	67 ^b^	137 ^b^	245 ^a^
Branched-chain fatty acids	31 ^b^	38 ^b^	67 ^a^
	Butyrate concentration		
Large intestine, mmol/kg digesta	8.6 ^b^	10.2 ^a^	13.3 ^a^
Mesenteric artery, μmol/L	2.8 ^c^	5.8 ^b^	8.1 ^a^
Portal vein, μmol/L	34 ^b^	75 ^b^	133 ^a^
Hepatic vein, μmol/L	6.3 ^b^	13.5 ^a^	17.2 ^a^

WSD, Western-style diet; RSD, resistant starch-enriched diet; AXD, arabinoxylan-enriched diet. ^a,b,c^ Mean values within a row with unlike superscript letters were significantly different (*p* ≤ 0.05). Modified from [19,49].

**Table 2:** Overview of effects of increased short-chain fatty acids and butyrate production and parameters related to intestinal inflammation.

Dietary Fibre Source	Species	Model	SCFA/Butyrate	Effects	Reference
AX + RS	Human	MetS	Faecal SCFA ↑ Faecal butyrate ↑	MCP1 ↓ IL-23A ↓ F-calprotectin ↓	[50,52]
Trans-GOS	Human	Overweight	Not measured	CRP ↓ Faecal calprotectin ↓	[93]
AX and RS	Pig	Healthy normal	Large intestinal SCFA pool size ↑ Large intestinal butyrate pool size ↑	NF-κB → MCP1 → TNFα →	[49,58]
Inulin	Rat	CRC	Faecal SCFA ↑ Faecal butyrate ↑	NF-κB ↓ IL-2 ↓ TNFα ↓ IL-10 ↓	[95]
*P. ovata* seeds	Rat	Colitis	SCFA production ↑ Butyrate production ↑	TNFα ↓ NO synthase ↓	[94]

SCFA, short-chain fatty acids; AX, arabinoxylan; RS, resistant starch; MetS, metabolic syndrome; MCP1, monocyte chemotactic protein 1; GOS, galactooligosaccharides; IL, interleukine; NF-κB, nuclear factor kappa-light-chain-enhancer of activated B cells; CRP, acute-phase C-reactive protein; TNFα, tissue necrotic factor α; CRC, colorectal cancer; NO, nitric oxide. ↑ ↓ → denote if the level is higher, lower or the same as in the control group.

**Table 3:** Overview of effects of increased short-chain fatty acids and butyrate production and parameters related to systemic inflammation.

Dietary Fibre Source	Species	Model	SCFA/Butyrate	Effects	Reference
AX + RS	Human	MetS	Faecal SCFA ↑ Faecal butyrate ↑	Hs-CRP → IL-6 → IL-1RA →	[51]
Trans-GOS	Human	Healthy elderly	Not measured	IL-6 ↓ IL-1β ↓ TNFα ↓ IL-10 ↑	[93]
FOS	Human	Elderly	Not measured	IL-6 (mRNA) ↓	[111]
RS	Human	MetS	Not measured	IL-6 → TNFα →	[118]
Whole grain rye and wheat vs. refined flour	Human	MetS	Not measured	Hs-CRP → IL-6 → IL-1RA → TNFα →	[117]
Whole grain	Human	Overweight, BMI > 25 kg/m^2^	Not measured	Hs-CRP → IL-6 →	[116]
High-fibre diet based on oat bran, rye bran and sugar beet fibre vs. low-fibre diet based on refined products	Human	Hypercholesterolemic subjects	Not measured	CRP ↓ IL-6 → IL-1RA → TFNα → IFN-γ → IL-17A → IL-1β → IL-7→	[115]
Healthy Nordic high-fibre diet vs. low-fibre refined control	Human	MetS	Not measured	Hs-CRP → IL-1RA ↓ IL-1β → IL-6 → IL-10 →	[113]
RS	Human	Prediabetic	Not measured	Hs-CRP → TNFα ↓ IL-6 →	[112]

SCFA, short-chain fatty acids; AX, arabinoxylan; RS, resistant starch; GOS, galactooligosaccharides; FOS, fructooligosaccharides; MetS, metabolic syndrome; BMI, body mass index; hs-CRP, high-sensitive acute-phase C-reactive protein; IL, interleukine; TNFα, tissue necrotic factor α. ↑ ↓ → denote if the level is higher, lower or the same as in the control group.
